# Clinical features and genetic mechanisms of anxiety, fear, and avoidance: A comprehensive review of five anxiety disorders

**DOI:** 10.1038/s41380-025-03155-1

**Published:** 2025-08-19

**Authors:** Kazutaka Ohi, Daisuke Fujikane, Kentaro Takai, Ayumi Kuramitsu, Yukimasa Muto, Shunsuke Sugiyama, Toshiki Shioiri

**Affiliations:** Department of Psychiatry, Gifu University Graduate School of Medicine, Gifu, Japan

**Keywords:** Psychiatric disorders, Diagnostic markers

## Abstract

Anxiety disorders, including social anxiety disorder (SAD), generalized anxiety disorder (GAD), panic disorder (PD), agoraphobia (AG), and specific phobia (SP), are characterized by core features of excessive anxiety and fear. Furthermore, these disorders are often accompanied by avoidance behaviors. While avoidance is a common behavioral response, it may be a consequence of or a cocontributor to the anxiety and fear driving these disorders. This narrative review integrates the interactions among anxiety, fear, and avoidance behaviors across these five disorders and synthesizes findings from clinical, neuropsychological, brain function, treatment, genetic, and epigenetic studies. Based on the intensity of anxiety and fear, these anxiety five disorders can be categorized into three groups: fear-dominant (SP, AG), mixed (PD, SAD), and anxiety-dominant (GAD). The intensity of avoidance behaviors is related to the age of onset. Each group responds to different treatment approaches. In fear-dominant disorders, exposure therapy is highly effective in reducing avoidance behaviors and fear responses. Cognitive behavioral therapy (CBT) with an emphasis on exposure is useful. In the mixed group, CBT is the preferred treatment, with a focus on both exposure therapy and cognitive restructuring. In addition to CBT, selective serotonin and serotonin-norepinephrine reuptake inhibitors (SSRIs/SNRIs) are commonly used to reduce anticipatory anxiety and fear symptoms. In anxiety-dominant disorders, both SSRIs/SNRIs and CBT with an emphasis on cognitive restructuring are effective for managing chronic worry. Anxiety and fear are regulated by distinct but interacting neurobiological mechanisms, with the amygdala central to fear processing and the hypothalamic‒pituitary‒adrenal axis involved in chronic anxiety regulation. Genetic and epigenetic studies demonstrate substantial heritability across anxiety disorders, with varying degrees of genetic influence on anxiety, fear, and avoidance. Avoidance behaviors, particularly in early-onset disorders such as SP and SAD, may be more strongly influenced by genetic factors. Large-scale genome-wide association studies (GWASs) grouping anxiety disorders have identified shared genetic loci, but GWASs for individual anxiety disorders are limited by small sample sizes. Grouping anxiety disorders into broader categories – namely, fear-dominant, mixed, and anxiety-dominant – rather than considering each specific anxiety disorder in isolation may lead to increased statistical power and yield more comprehensive perspectives on the shared and distinct clinical and genetic risk factors among anxiety disorders.

## Introduction

Anxiety disorders are a group of mental health disorders characterized by excessive fear, anxiety, and related behaviors such as avoidance. These disorders are highly prevalent worldwide, with a lifetime prevalence of approximately 30% [1–3]. Anxiety disorders can be categorized as follows: social anxiety disorder (SAD), generalized anxiety disorder (GAD), panic disorder (PD), agoraphobia (AG), and specific phobia (SP). Although obsessive-compulsive disorder (OCD) and post-traumatic stress disorder (PTSD) exhibit some overlapping features with anxiety disorders, they are not classified within this category and are therefore excluded from this review. The primary reason for their exclusion is that both OCD and PTSD are categorized separately from anxiety disorders in current international diagnostic criteria (DSM-5) due to their distinct neurobiological and clinical characteristics [4, 5]. OCD is characterized by intrusive obsessive thoughts and compulsive behaviors, which are primarily driven by distress and cognitive rigidity rather than by fear and avoidance, as seen in anxiety disorders. PTSD, on the other hand, is primarily defined by trauma-related re-experiencing, hyperarousal, and emotional numbing, distinguishing it from the fear-anxiety spectrum of traditional anxiety disorders. Neurobiological studies further suggest that OCD involves dysfunctions in the cortico-striato-thalamo-cortical (CSTC) circuitry [6, 7], while PTSD is linked to impaired fear extinction mechanisms in the amygdala-prefrontal network [8]. Additionally, distinct epigenetic modifications, particularly DNA methylation, may regulate gene expression in these disorders [9]. Given these distinctions, this review focuses specifically on the five anxiety disorders traditionally categorized within the anxiety spectrum: SAD, GAD, PD, AG, and SP. Each anxiety disorder presents a unique profile of symptoms, but they share common features of heightened anxiety, fear, and avoidance behaviors.

SAD is characterized by a pronounced fear of social or performance situations, where individuals worry about being negatively judged or observed by others. This fear drives avoidance behaviors such as avoiding public speaking or social gatherings, which can severely impair social and occupational functioning [10]. GAD is characterized by chronic, excessive worry about various topics, often without a clear trigger. The persistent worry leads to generalized feelings of tension and apprehension, often affecting multiple areas of daily life [11]. PD is associated with recurrent, unexpected panic attacks, accompanied by intense fear and physical symptoms such as heart palpitations, sweating, and dizziness. The anticipatory anxiety of having another attack often contributes to the development of AG, where individuals avoid places or situations where escape might be difficult or where help might not be available [12]. AG often presents as the avoidance of open spaces, crowded areas, or public transportation [13]. Finally, SP involves intense fear of a specific object or situation, such as heights, animals, or flying. Exposure to a feared stimulus triggers immediate fear responses, which often result in avoidance behaviors [14]. Although these disorders present distinct patterns of symptoms, such as varying intensities of fear and anxiety, different ages of onset [1], and unique behavioral manifestations, these anxiety disorders share the core features of heightened anxiety and fear, often accompanied by avoidance behaviors.

This narrative review integrates current knowledge on the interactions among anxiety, fear, and avoidance behaviors across these five anxiety disorders. On the basis of the intensity of fear, anxiety, and avoidance behaviors, we can categorize these five anxiety disorders into three distinct groups: fear-dominant (SP, AG), mixed (PD, SAD), and anxiety-dominant (GAD). This review synthesizes findings from clinical, neuropsychological, brain function, treatment, genetic, and epigenetic studies, with a particular focus on how these components across fear, anxiety and avoidance behaviors contribute to disorder-specific profiles.

## Anxiety, fear, and avoidance: conceptual framework

### Core differences between anxiety and fear

Anxiety and fear are related but conceptually distinct states. Fear is an acute emotional response to a real or perceived immediate threat, activating the autonomic nervous system and preparing the body for a fight-or-flight reaction [3, 15, 16]. In contrast, anxiety is a more diffuse, future-oriented state of unease or worry without a specific external stimulus. The primary distinction between the two lies in their temporal orientation: fear is reactive to immediate danger, whereas anxiety is anticipatory and concerned with potential future threats [3, 15]. On the basis of three responses, namely, verbal-subjective, overt motor acts, and somato-visceral activity [15], fear is characterized by thoughts of imminent threat, urges to escape, and a strong autonomic surge resulting in physical symptoms such as sweating, trembling, heart palpitations, and nausea, whereas anxiety is often expressed through worry, avoidance, and muscle tension.

Several studies support the distinction between fear and anxiety on the basis of self-reported symptoms. A two-factor model, which distinguishes between physiological arousal (e.g., heart racing) and subjective anxiety (e.g., nervousness, inability to relax), has demonstrated a better fit than a one-factor model across diverse populations, including children/adolescents, undergraduates, and psychiatric outpatients [17–19]. These findings indicate that fear is more closely associated with autonomic arousal, whereas anxiety is linked to subjective cognitive distress. However, while fear and anxiety are conceptually distinct, the symptoms of these states exist along a continuum and are likely to diverge and converge to varying degrees. Clinically, anxiety disorders can lean toward fear (e.g., AG) or anxiety (e.g., GAD), but these disorders frequently overlap. Anxiety can heighten fear responses, while fear can contribute to persistent anxious states, reinforcing a cycle of avoidance and distress.

### Avoidance behavior: consequence or co-contributor?

Avoidance behavior, a defensive response, is a hallmark of anxiety disorders, serving to temporarily reduce the distress caused by fear or anxiety. However, this avoidance paradoxically reinforces anxiety and fear by preventing exposure to the feared stimulus, perpetuating the anxiety disorder [3, 20]. Avoidance manifests in various forms, ranging from the avoidance of social situations by individuals with SAD to the avoidance of open spaces by individuals with AG; furthermore, the severity of avoidance behaviors is correlated with both the chronicity and overall severity of anxiety disorders [3].

Avoidance behavior is also associated with poorer treatment outcomes, particularly in SP, where high levels of avoidance predict lower responsiveness to exposure-based therapy [21]. Additionally, avoidance behaviors are related to an earlier onset in certain anxiety disorders, such as SAD and PD, where avoiding feared stimuli can lead to earlier development of chronic symptoms [10, 22]. Early-onset anxiety disorders, including SP and SAD, often emerge during critical developmental periods such as childhood or adolescence, potentially disrupting socialization and academic progress, and establishing persistent avoidance patterns that extend into adulthood [23].

In contrast, later-onset disorders such as GAD tend to develop during adulthood, potentially resulting in fewer habitual avoidance patterns [22, 24]. Although avoidance is typically considered a consequence of anxiety and fear [25], the evidence suggests that it may also act as a cocontributor, maintaining or exacerbating anxiety through a cycle of negative reinforcement [26, 27]. This feedback loop inhibits habituation to the feared stimuli, prolonging and intensifying anxiety over time.

### Clinical features of five anxiety disorders

**Fig. 1:**
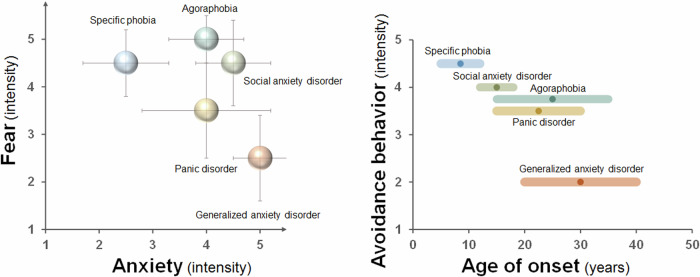
Relationships between the intensity of anxiety, fear, and avoidance behaviors and the age of onset for each anxiety disorder. On the basis of previous studies and our expert consensus, intensities (bubbles) and variations (error bars) of anxiety and fear are displayed across different anxiety disorders. The median age of onset, along with its range, is indicated for each disorder.

Figure 1 illustrates the relationships between the intensities of fear and anxiety across five anxiety disorders, as well as the relationships between the intensity of avoidance behaviors and the age of onset for each disorder, based on previous studies (established diagnostic criteria and assessment scales) and our expert consensus. These intensities represent typical assessments but may vary depending on the clinical setting and individual patient experience. These values may serve as useful general references rather than absolute consensus indicators.

#### SAD

##### Fear

Very high (★★★★☆), particularly in situations involving potential social rejection or when a negative evaluation is anticipated [10]. The fear is typically focused on social interactions or performance situations.

##### Anxiety

High and persistent (★★★★☆), often occurring well in advance of the feared event. Individuals may experience anticipatory anxiety days or weeks before the actual event [28].

##### Avoidance

Significant (★★★★☆), leading individuals to avoid any situation where they fear being judged. This avoidance can severely affect social and occupational functioning. The age of onset for SAD is typically early adolescence, around age 13, and avoidance behaviors may become deeply ingrained if left untreated [22, 29].

#### GAD

##### Fear

Low (★★☆☆☆), lower than in other anxiety disorders. Fear in GAD patients is often not tied to specific triggers but is generalized to involve multiple domains, such as health, finances, or safety [11].

##### Anxiety

Extremely high and chronic (★★★★★), affecting multiple areas of life. Individuals experience excessive uncontrollable worry, which affects more days than not over a period of six months [11].

##### Avoidance

Mild (★★☆☆☆), less pronounced than other anxiety disorders, although individuals may avoid situations that heighten their worries, such as stressful tasks or conflict situations. The onset of avoidance among individuals with GAD typically occurs in early adulthood, often in the early 30 s [2], with avoidance being less prominent but growing over time as a coping mechanism for chronic worry [30].

#### PD

##### Fear

High (★★★☆☆ to ★★★★☆), extremely high during panic attacks. Individuals often experience intense fear of losing control, dying, or going insane [31].

##### Anxiety

High anticipatory anxiety (★★★★☆), with individuals fearing the occurrence of future panic attacks, which can become as disabling as the attacks themselves [31].

##### Avoidance

Moderate to high (★★★☆☆ to ★★★★☆). Individuals begin to avoid places or situations where they have experienced panic attacks, fearing recurrence. This avoidance can lead to the development of AG, where individuals may avoid leaving their homes altogether [32]. The onset of avoidance among individuals with PD typically ranges between late adolescence and early adulthood, around the ages of 20–24 years [2].

#### AG

##### Fear

Extremely high (★★★★★), particularly in situations where individuals perceive that they cannot escape or receive help during a panic attack. Common fears include being in crowds, open spaces, or public transportation [13].

##### Anxiety

High and pervasive (★★★★☆), not only in feared situations but also in anticipation of entering these environments.

##### Avoidance

High (★★★★☆), with individuals often becoming housebound, severely limiting their social and occupational functioning [33]. The onset of avoidance among individuals with AG typically occurs between late teens and mid-30s; however, AG may also develop following PD [2].

#### SP

##### Fear

Extremely high (★★★★☆ to ★★★★★), directly tied to a specific object or situation (e.g., heights, animals, flying). Exposure to a phobic stimulus triggers immediate fear responses, often resulting in panic-like symptoms [14].

##### Anxiety

Moderate anxiety (★★☆☆☆ to ★★★☆☆), which is generally low in the absence of the phobic stimulus but can escalate to high levels when the feared object or situation is anticipated or encountered [14].

##### Avoidance

Very high (★★★★☆ to ★★★★★), with individuals going to great lengths to avoid the phobic stimulus, leading to significant lifestyle restrictions and a reduced quality of life. The onset of avoidance among individuals with SP is typically in childhood, and earlier onset often leads to more ingrained avoidance behaviors over time [22].

### Classification of anxiety disorders into three categories based on anxiety, fear, and avoidance behaviors: neuropsychological, brain function, and treatment implications

The five anxiety disorders can be categorized into three groups on the basis of the intensity of fear, anxiety, and avoidance behaviors: fear-dominant anxiety disorders, mixed anxiety disorders, and anxiety-dominant anxiety disorders. The fear-dominant group (SP, AG) is characterized by extremely high fear and high avoidance, where specific stimuli or open spaces trigger severe fear responses and heightened avoidance behaviors. The mixed group (PD, SAD) involves both high levels of fear and anxiety, with moderate to high avoidance. Panic attacks and fear of social rejection are key features that lead to avoidance in specific situations. The anxiety-dominant group (GAD) is characterized by chronic, pervasive anxiety with relatively low fear and less pronounced avoidance behaviors.

This classification aligns conceptually with the Hierarchical Taxonomy of Psychopathology (HiTOP), a dimensional framework that organizes mental disorders based on empirical data, including factor analysis, symptom co-occurrence patterns, and clinical research findings, rather than traditional diagnostic categories [34]. In the HiTOP model, anxiety disorders fall within the internalizing spectrum, which is further divided into a “fear” subfactor (e.g., SP, SAD, PD) and a “distress” subfactor (e.g., GAD) [34]. Our classification corresponds to these distinctions, with fear-dominant disorders reflecting the fear subfactor, anxiety-dominant disorders aligning with the distress subfactor, and mixed disorders incorporating elements of both. However, our classification uniquely emphasizes avoidance behaviors, which are not explicitly defined in HiTOP but may be crucial for understanding the persistence and treatment response of anxiety disorders. By incorporating avoidance as a key factor, this framework may provide additional insights into the clinical course, treatment strategies, and genetic underpinnings of these disorders.

While previous studies have categorized anxiety disorders based on symptom dimensions (e.g., fear vs. distress in HiTOP), the specific three-group classification, with an emphasis on avoidance behaviors, is unique to this study. To our knowledge, no prior review has explicitly proposed a classification that integrates fear, anxiety, and avoidance into a single framework. This novel approach may have important implications for understanding clinical heterogeneity and treatment responses in anxiety disorders.

### Neuropsychological findings across anxiety groups

Attentional control deficits, cognitive biases, and memory distortions contribute to the development and persistence of anxiety disorders, manifesting differently across anxiety groups (Table 1). In the fear-dominant group (SP, AG), individuals exhibit heightened attentional control deficits and biases toward threat-related stimuli and difficulty disengaging from threat cues, reinforcing avoidance behaviors [35]. SP involves excessive attentional capture by phobic objects, while AG patients exhibit hypervigilance toward inescapable cues. In the mixed group (PD, SAD), cognitive biases and distorted threat processing play a central role. PD is characterized by distorted interoceptive processing, leading to catastrophic misinterpretations of bodily sensations and exacerbating panic symptoms [36]. SAD involves excessive self-focused attention and negative interpretation biases, reinforcing fear of social evaluation and avoidance [37]. In the anxiety-dominant group (GAD), memory distortions, cognitive inflexibility, and impaired attentional control contribute to chronic worry. Individuals recall threat-related information more readily and overgeneralize past negative experiences, while deficits in inhibitory control and attentional shifting perpetuate anxious thought cycles [38, 39].

**Table 1:** Therapeutic recommendations and associated neuropsychological and brain functions across three anxiety disorder groups on the basis of intensities of fear, anxiety, and avoidance behaviors.

Anxiety group	Neuropsychological Findings	Brain Function	CBT (Exposure therapy)	CBT (Cognitive restructuring)	SSRIs/SNRIs	Additional therapies
Fear-dominant (SP, AG)	Heightened threat-related attentional biases and impaired disengagement from threat cues	Amygdala hyperactivity (exaggerated fear response), vmPFC dysfunction (reduced fear inhibition)	The primary intervention (++++), essential for reducing avoidance behaviors	Moderate (++), effective when combined with exposure for AG	Adjunctive, primarily for comorbid conditions (+)	None specific
Mixed (PD, SAD)	Distorted interoceptive processing (PD) and excessive self-focused attention with negative interpretation biases (SAD)	Amygdala-hippocampus hyperactivity (persistent fear memories), dlPFC dysfunction (reduced control of anticipatory anxiety)	Recommended (+++), effective in addressing avoidance	Highly recommended (++++) but with a stronger focus on avoidance reduction in PD and SAD	Highly recommended (++++), alongside CBT for managing anticipatory anxiety and panic symptoms	Gradual exposure and cognitive restructuring to reduce avoidance
Anxiety-dominant (GAD)	Negative memory biases, cognitive inflexibility, and impaired attentional control	HPA axis hyperactivation (chronic stress response), dmPFC dysfunction (reduced control of worry and rumination)	Less common (+), not the primary intervention but used occasionally	Essential (+++++), focusing on restructuring to manage chronic worry	Essential (+++++), often a cornerstone for long-term management of chronic worry	Mindfulness-based therapy (++) for reducing chronic worry

*SP* specific phobia; *AG* agoraphobia; *PD* panic disorder; *SAD* social anxiety disorder; *GAD* generalized anxiety disorder; *CBT* cognitive behavioral therapy; *vmPFC* ventromedial prefrontal cortex; *dlPFC* dorsolateral prefrontal cortex; *dmPFC* dorsomedial prefrontal cortex; *HPA* hypothalamic‒pituitary‒adrenal; *SSRIs* selective serotonin reuptake inhibitors; *SNRIs* serotonin‒norepinephrine reuptake inhibitors.

### Brain function across anxiety groups

Meta-analyses of brain structures have demonstrated that a reduced volume of the ventral anterior cingulate gyrus and inferior frontal gyrus is common among individuals with anxiety disorders [40]. Moreover, another study showed that GAD patients exhibit disorder-specific altered volumes relative to fear-related anxiety disorders, including decreased volumes in the insula and lateral/medial prefrontal cortex (PFC), as well as increased volumes in the putamen [41]. These findings suggest distinct neural substrates underlying different anxiety disorder groups.

**Fig. 2:**
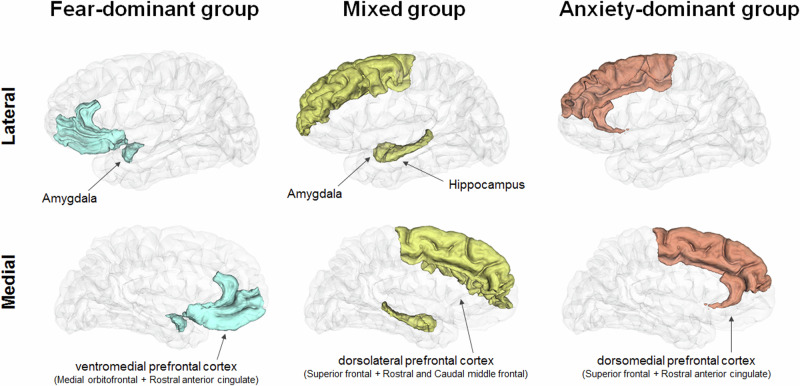
Brain function related to three anxiety disorder groups: fear-dominant (SP, AG), mixed (SAD, PD), and anxiety-dominant (GAD), based on the intensities of fear, anxiety, and avoidance behaviors. In the anxiety-dominant group, the hypothalamic‒pituitary‒adrenal (HPA) axis has also been implicated. SP, specific phobia; AG, agoraphobia; PD, panic disorder; SAD, social anxiety disorder; GAD, generalized anxiety disorder.

In contrast, each anxiety group involves distinct brain functions (Fig. 2 and Table 1). In the fear-dominant group (SP, AG), hyperactivity in the amygdala leads to exaggerated fear responses. The ventromedial PFC, which is typically involved in processing fear and risk, shows reduced inhibitory control over the amygdala, contributing to heightened fear responses [20]. In the mixed group (PD, SAD), the dorsolateral PFC (dlPFC) plays a crucial role in executive function and cognitive control, particularly in modulating emotional responses. Dysfunction in connectivity between the amygdala and hippocampus in this group contributes to the persistence of fear-related memories [42]. Additionally, the dlPFC is responsible for managing anxious thoughts, and its dysfunction is associated with impaired emotional regulation [43, 44]. In the anxiety-dominant group (GAD), the dorsomedial PFC (dmPFC) is implicated in anxiety regulation. The dmPFC is involved in monitoring and controlling emotional responses to perceived threats. Chronic activation of the hypothalamic‒pituitary‒adrenal (HPA) axis leads to prolonged cortisol release, exacerbating anxiety symptoms [45].

### Treatment implications by anxiety groups

The classification of anxiety disorders into fear-dominant, mixed, and anxiety-dominant groups directly informs treatment strategies (Table 1). International treatment guidelines provide consensus-based recommendations, emphasizing cognitive behavioral therapy (CBT) as the first-line treatment, followed by pharmacotherapy when necessary. The German guidelines for anxiety disorders recommend CBT as the psychological treatment with the highest level of evidence, with selective serotonin reuptake inhibitors (SSRIs) or serotonin-norepinephrine reuptake inhibitors (SNRIs) as first-line pharmacological options. Benzodiazepines are discouraged due to dependency risks, while treatment-resistant cases may benefit from switching antidepressants or combination therapy [46]. Similarly, the Royal Australian and New Zealand College of Psychiatrists (RANZCP) clinical practice guidelines recommend CBT and SSRIs/SNRIs as standard treatments, with combination therapy considered for severe cases [47].

In the fear-dominant group (SP, AG), exposure therapy is highly effective [48, 49]. Gradually exposing patients to feared stimuli helps reduce exaggerated fear responses and avoidance behaviors [50, 51]. CBT, which involves exposure, is beneficial for SP but focuses primarily on the behavioral (exposure) component rather than on cognitive restructuring. For AG, CBT combines cognitive restructuring and exposure therapy, helping patients challenge their fears and reduce avoidance [52]. While SSRIs and SNRIs are not typically recommended for SP, they may be used to treat AG when comorbid with other anxiety disorders [31]. However, some individuals remain resistant to standard treatments, requiring alternative therapeutic strategies.

In the mixed group (PD, SAD), CBT is the preferred treatment. It focuses on both exposure therapy and cognitive restructuring [10, 31, 53]. CBT has demonstrated strong efficacy across both PD and SAD, reducing avoidance and fear responses. In SAD patients, CBT is particularly effective in addressing fear of negative evaluation and social avoidance. In PD, CBT helps patients correct catastrophic misinterpretations of panic symptoms and encourages gradual exposure to avoided situations. In addition to CBT, SSRIs/SNRIs serve as first-line pharmacological treatments, targeting both anticipatory anxiety and acute fear responses [10, 31]. However, residual symptoms persist in some patients, necessitating augmentation strategies and alternative interventions.

In the anxiety-dominant group (GAD), SSRIs/SNRIs are frequently used for long-term management of chronic anxiety [54, 55]. CBT is also highly effective and focuses on cognitive restructuring to reduce chronic worry and somatic symptoms. Behavioral strategies, such as exposure to uncertainty, are key components of CBT in individuals with GAD [56]. However, owing to the nature of GAD, which involves generalized and ongoing worry, CBT may require more sessions than other anxiety disorders do [52]. Mindfulness-based therapy have also gained support as adjunctive treatments, encouraging patients to focus on the present moment and reducing excessive worry [57].

Despite the effectiveness of CBT and pharmacotherapy, some patients experience residual symptoms, relapse, or treatment resistance. SSRIs and SNRIs, while first-line pharmacological treatments, have limitations such as delayed onset, side effects, and partial response rates. Similarly, CBT, though highly effective, requires patient motivation and access to trained therapists. For treatment-resistant cases, alternative approaches are under investigation. Deep brain stimulation (DBS) is approved for treatment-resistant OCD in some countries, targeting circuits such as the ventral capsule/ventral striatum and subthalamic nucleus to reduce symptom severity [58]. However, its invasiveness, high cost, and limited long-term data restrict broader clinical application in anxiety disorders. Non-invasive neuromodulation techniques, including transcranial magnetic stimulation (TMS) and transcranial direct current stimulation (tDCS), are being explored for treatment-resistant anxiety disorders [59–62]. TMS modulates prefrontal activity via electromagnetic pulses, while tDCS alters neuronal excitability through electrical currents. Though promising, further research is needed to optimize protocols and assess long-term efficacy. These findings highlight the need for personalized approaches and novel interventions, with neuromodulation offering a potential future avenue for patients who do not respond to conventional treatments.

### Genetics related to three anxiety groups

**Fig. 3:**
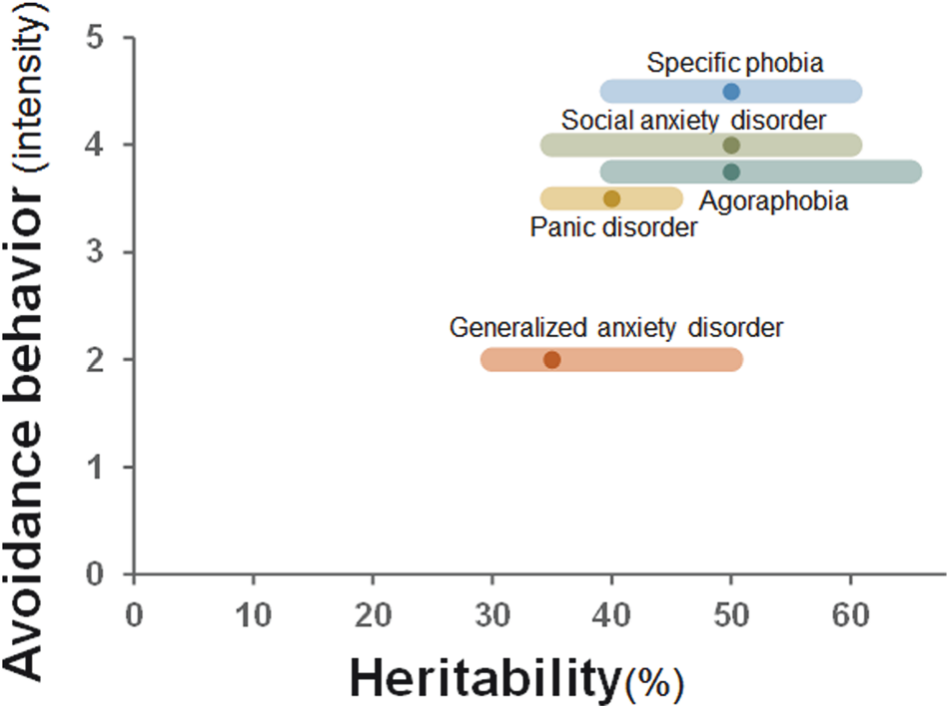
Relationships between the intensity of avoidance behaviors and the heritability for each anxiety disorder. Mean heritability, along with its range, is shown for each disorder, positioned according to the intensity of avoidance behaviors.

Twin and family studies have consistently shown that genetic factors significantly contribute to the development of anxiety disorders, although the degree of heritability varies across the five disorders [63] (Fig. 3). SAD has heritability rates ranging from approximately 40% to 60% [63–67], with evidence suggesting that heritability may change with age and that SAD aggregates in families. GAD is also moderately heritable, with estimates ranging from 30% to 50% [63, 68]. Family studies indicate that GAD shares genetic factors with depression and other anxiety disorders. PD shows a heritability estimate of approximately 40% [63, 65, 69], with family studies highlighting an increased risk of PD, other anxiety disorders, and depression in first-degree relatives [70]. AG shares genetic risk factors with PD [71], and twin studies estimate that AG heritability [65, 72, 73]. For SP, twin studies suggest heritability rates between 40% and 60% [65, 73]. However, heritability can vary on the basis of the type of phobia, with blood–injury–injection phobia showing higher heritability (60%) than animal or situational phobias (45%) [72, 73].

Among fear, anxiety, and avoidance behaviors, genetic factors play a substantial role across all three traits. Avoidance behaviors show the strongest heritability, followed by fear [63, 74–76]. While avoidance behaviors may be secondary to fear and anxiety, early-onset anxiety disorders such as SP and SAD suggest that avoidance behaviors may be more strongly influenced by genetic factors (Fig. 3). These behaviors often emerge early in development and are central to the progression of the disorder. Across anxiety groups, a gradient of genetic involvement is evident: fear-dominant disorders tend to show a higher degree of heritability [74, 75] than mixed and anxiety-dominant disorders (Fig. 3). Although fear and anxiety may share some overlapping genetic factors, previous studies have suggested that fear and anxiety have distinct biological mechanisms [65, 77]. The amygdala plays a central role in fear processing [78, 79], whereas the HPA axis and serotonergic regulation are more involved in chronic stress and anxiety [80]. These findings support the hypothesis that different genetic pathways may influence the specific manifestations of anxiety disorders. PD and SAD fall into a mixed category where genetic influences from both fear and anxiety come together. This finding aligns with neurobiological evidence showing that while a genetic gradient exists, fear and anxiety share some genetic origins but also have distinct differences.

In recent years, genetic research on common diseases has focused primarily on genome-wide association studies (GWASs), remarkably advancing our understanding of complex traits, including anxiety disorders. For PD, a large-scale GWAS conducted by the Psychiatric Genomics Consortium (PGC) included 2248 PD patients and 7992 controls. However, no genome-wide significant loci were identified, likely because of the small sample size [81]. For other anxiety disorders, such as SAD, SP, GAD, and AG, large-scale GWASs are still lacking. To address the challenge of limited sample sizes for individual anxiety disorders, several consortia have conducted three large-scale case‒control GWASs, pooling samples from five anxiety-related disorders: the Anxiety NeuroGenetics STudy (ANGST) [82], iPSYCH [83], and UK Biobank (UKBB) [84] studies, with sample sizes ranging from 3695 to 25,453 anxiety cases and 13,615 to 58,113 controls. Additionally, a large-scale GWAS of a continuous anxiety trait, using the Generalized Anxiety Disorder 2-item scale (GAD-2), was conducted in 199,611 individuals by the Million Veteran Program [85]. These combined approaches, aimed at increasing statistical power, have identified 1 to 5 genome-wide significant loci shared across anxiety disorders. Recently, the largest and most recent GWAS of anxiety disorders identified 58 genome-wide significant loci in 122,341 anxiety cases and 729,881 controls [86].

LD score regression (LDSC) analyses have demonstrated significant genetic correlations across the GWASs of anxiety disorders (*r*_*g*_ > 0.70) [84], indicating substantial genetic overlap among anxiety disorders. Although the GWAS of PD also showed significant genetic overlap with broader anxiety disorder GWASs, the correlation was somewhat lower (*r*_*g*_ ≈ 0.60) [81]. This suggests that while PD shares some common genetic factors with other anxiety disorders [65, 77], it may also have distinct genetic determinants. These findings emphasize the importance of conducting large-scale, disorder-specific GWASs for each anxiety disorder to further elucidate their genetic backgrounds and to identify both unique and shared genetic risk factors across anxiety disorders.

### Epigenetics related to three anxiety groups

Beyond genetic predispositions, epigenetic modifications, particularly DNA methylation, play a key role in the development and regulation of anxiety disorders [9, 87–90]. Epigenetic mechanisms regulate gene expression without altering the DNA sequence, providing a molecular link between genetic susceptibility and environmental influences. Recent epigenome-wide association studies (EWASs) have identified distinct DNA methylation patterns associated with anxiety disorders, including SAD, PD, and GAD, compared to controls [9, 87–89].

A large-scale EWAS involving 618 individuals with anxiety disorders and 514 controls identified replicable differentially methylated positions (DMPs) in genes potentially implicated in psychiatric disorders. Notably, the overlapping EWAS signals between discovery and replication cohorts were enriched for genetic variants previously identified in GWAS of anxiety disorders, major depressive disorder (MDD), and PTSD [87]. Additionally, an EWAS examining 66 individuals with SAD and 77 healthy controls investigated the impact of early-life adversity, revealing DMPs related to PD and DNA methylation differences associated with early-life stress. These findings suggest that environmental factors may contribute to epigenetic modifications underlying SAD [88]. Regarding GAD, an EWAS of whole blood samples from 93 GAD drug-naïve, first-episode patients, 65 OCD patients, and 302 controls identified disorder-specific epigenetic alterations. However, this study did not directly compare GAD patients with controls, instead focusing on differentiating GAD from OCD [9]. Furthermore, a study investigating the effects of stressful life events on DNA methylation in 183 PD patients, 102 MDD patients, and 85 healthy controls demonstrated that environmental stress can induce epigenetic changes, potentially contributing to age acceleration through widespread dysregulation of physiological systems [89]. Additionally, methylation risk scores, derived from cumulative effects of genome-wide DNA methylation, were found to be associated with anxiety disorder susceptibility. Specifically, methylation risk scores for SAD were linked to an increased risk of PD, while methylation risk scores for stressful life events in PD were paradoxically associated with a lower risk of SAD, possibly due to avoidance behavior [90].

Taken together, these findings suggest that both shared and disorder-specific DNA methylation profiles may contribute to the three anxiety groups. However, large-scale EWASs, such as PGC Anxiety Epigenetics Working Group, are needed to validate and enhance these findings due to the limited sample sizes in these studies.

## Conclusion

The relationships among anxiety, fear, and avoidance behaviors across the five anxiety disorders—SAD, GAD, PD, AG, and SP—are complex. Understanding these relationships is crucial for elucidating the clinical features, neuropsychological processes, brain functions, and genetic and epigenetic mechanisms underlying anxiety disorders. Anxiety and fear share overlapping pathways, including brain circuits such as the amygdala for fear processing and the HPA axis and serotonergic regulation for anxiety. However, the unique genetic and epigenetic contributions to these behaviors require further investigation. Avoidance behaviors, particularly in early-onset disorders such as SP and SAD, may be more strongly influenced by genetic factors, contributing to their persistence and treatment resistance.

Given that conducting GWASs for individual anxiety disorders often results in small sample sizes, limiting the ability to detect significant genetic loci, a more effective approach may be to conduct GWASs by grouping anxiety disorders into three distinct anxiety categories: fear-dominant, mixed, and anxiety-dominant. Grouping disorders in this way increases sample sizes, improves statistical power, and facilitates the identification of shared genetic and epigenetic variants across disorders. Furthermore, this approach facilitates the investigation of genetic correlations with other psychiatric disorders, such as depression, OCD, and PTSD, and intermediate phenotypes, such as neuroticism and emotional regulation. By integrating behavioral, neuropsychological, brain function, treatment, genetic, and epigenetic insights, we can gain a more comprehensive understanding of the broader biological and clinical frameworks underlying anxiety disorders, thus clarifying both their shared and unique features across anxiety disorders.

## Data Availability

The data will be made available upon request.
